# Supplementary material for: Rise and Recharge: Exploring Employee Perceptions of and Contextual Factors Influencing an Individual-Level E-Health Smartphone Intervention to Reduce Office Workers’ Sedentary Time at Work
Source: Int J Environ Res Public Health. 2021 Sep 13;18(18):9627. doi: 10.3390/ijerph18189627 (PMC8467510; doi:10.3390/ijerph18189627)

Supplementary materials

Image: Supplementary materials 1: Ecological Momentary Assessment questionnaires

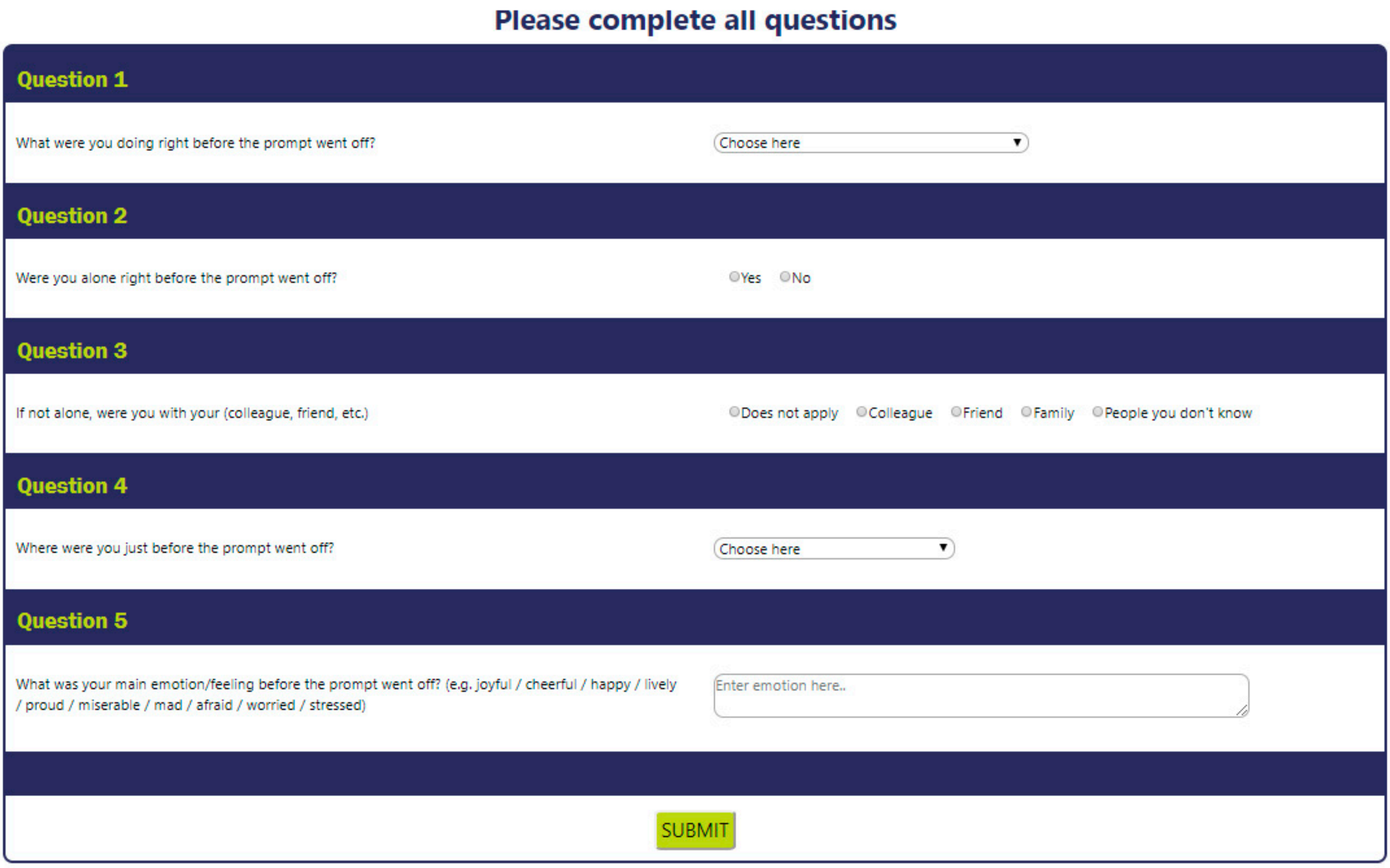

Please complete all questions

Question 1

What were you doing right before the prompt went off? Choose here

Question 2

Were you alone right before the prompt went off? Yes No

Question 3

If not alone, were you with your (colleague, friend, etc.) Does not apply Colleague Friend Family People you don't know

Question 4

Where were you just before the prompt went off? Choose here

Question 5

What was your main emotion/feeling before the prompt went off? (e.g. joyful / cheerful / happy / lively / proud / miserable / mad / afraid / worried / stressed) Enter emotion here..

SUBMIT

a) Prompt Form

b) Review Form

Question 1

How much did you break up your sitting this week compared to the week before the intervention started?

0 1 2 3 4 5 6 7 8 9 10
Not at all ○ ○ ○ ○ ○ ○ ○ ○ ○ ○ ○ All of the time

Question 2

How often did you engage with the prompts this week?

0 1 2 3 4 5 6 7 8 9 10
Not at all ○ ○ ○ ○ ○ ○ ○ ○ ○ ○ ○ All of the time

Question 3

Why did you give it this score?

Enter reason here..

Question 4

What stopped or encouraged you to engage?

Enter reason here..

Question 5

If no, what stopped you doing so?

Enter reason here..

Question 6

How likely are you to engage with the prompts next week?

0 1 2 3 4 5 6 7 8 9 10
Not at all ○ ○ ○ ○ ○ ○ ○ ○ ○ ○ ○ All of the time

Question 7

Why do you think this is?

Enter reason here..

Question 8

How helpful do you find the prompts?

0 1 2 3 4 5 6 7 8 9 10
Not at all ○ ○ ○ ○ ○ ○ ○ ○ ○ ○ ○ All of the time

Question 9

Any comments on the app at this stage?

Enter reason here..

SUBMIT

Text: Supplementary materials 1. Rise and Recharge focus group schedule

Semi- structured focus group schedule- 12-week data collection

Facilitator introduction:

You have been asked to take part in this focus group due to your participation in the Rise and Recharge workplace intervention over the last 12 weeks. We would like to take this opportunity to explore the factors influencing whether you were able to adhere to the prompt to break up you r sitting time every 30 or every 60 minutes during working hours and explore whether these factors are consistent or contrary between your various worksites/ locations job roles.

My role here today is to facilitate and guide a discussion about the Rise and Recharge intervention. I will be asking you questions about your experiences and perceptions and I may prompt you for more detailed information or to clarity meaning through if needed.

Please be aware that there are no right or wrong answers to the questions today. You may have different experiences or points of view to others, so please feel free to share your point of view even if it differs from what others have said. Keep in mind that we are equally interested in positive and negative comments, and at times the negative comments can be the most informative and will help us improve the project if it is delivered again for others.

Please allow each person to voice their opinion and please avoid talking over each other. I am tape recording this session because I don't want to miss any of your comments and feedback. We will be on a first name basis today, however we won't use any names in our reports. Everything will be kept confidential and you will be anonymized during transcription. The focus groups should take no longer than 40 minutes.

So to recap. You are taking part in the rise and recharge intervention and you are in the (Intervention- prompt/ control- no prompt) arm. So far you have completed three health checks and have been involved in the intervention for 12 weeks. I will be asking you to reflect on the times during the intervention when you received a prompt to break up your sitting time. I would like to explore whether you were, or were not able to break up your sitting time and what the factors were surrounding this.

- **Icebreaker post-it note activity**: Can you think of one example when you received a prompt during working hours- you can pick an example of when you did or did not adhere to the prompt. Can you answer the following;
    - Who you were with? What you were doing? When you received the prompt? Where you were? Why you did or did not adhere to the prompt?

1. How did you feel at the time when the prompt went off?
2. Can you describe your experience of taking part in the Rise and Recharge intervention over the last 12 weeks?
3. Were there any factors that prevented you from adhering to the Rise and Recharge prompts at work?
    a. How frequently did you experience this?
    b. Can you envisage any strategy/factors that would support you to overcome this barrier?
4. Where there any factors that encouraged/motivated you to adhere to the Rise and Recharge prompts at work?
    a. Who/ What/ when /where/ why/ how
    b. How frequently did you experience this?
5. Can you give any examples of any strategies that you employed to help you to break up your sitting time at work?
6. Is there anything that could support you to break up your sitting time more frequently at work?
7. Has your experience of the intervention impacted (positively or negatively) on your:
    a. Health (physical/ mental)/ Performance at work/ any unexpected consequences?
8. Did the use of the app have any impact (positive or negative) on you behaviour outside of work?
9. How user friendly was the e-health application to use?
10. Can you give an example of what you liked/disliked about the e-health application? How could it be improved?
11. How did you feel about the frequency of the prompts?
12. How likely would you be to continue using the e-health application in the future and why?

Table: Supplementary materials 1: Thematic analysis framework

Theme	Descriptor	Ecological level code	Supporting quotation
Health driven motivation for participation	Desire to understand about individual health through regular intervention health checks.	Interpersonal	"[…] *So I was just curious as to how the intervention may help me actually sort of move around a bit more and what impact that may or may not have on my day to day work and my health as well*." P04 C1 Prompt 30<br>*"I wanted to get involved for selfish reasons really to find out my sort of cholesterol levels and blood sugar, blood glucose, because there is a history of heart disease and diabetes in my family so I thought it'll be a good opportunity to find out where I stand with that and whether breaking up my sitting patterns in work would, you know, indeed help with that as well."* P51 C1 Prompt 60
Peer support as driver for participation and engagement	Working in an office with peers/ colleagues taking part in the intervention helped encourage ongoing participation.	Intrapersonal	*"At the beginning you know I quite often when I saw other people on the team standing up, move around and do this that and the other, I thought oh this could be quite a good idea, because sometimes you can sit there for 3 hours*." P55 C1 Prompt 30
Facilitators	Increased health awareness among colleagues	Intrapersonal	*"I think* [the intervention has] *made* [breaking up sitting time] *less of an odd behavior. Before, if someone stood up you'd think 'oh why is he stood up to do his skype call' whereas now maybe the whole idea of standing up at your desk is not that weird. So it's acceptable"* P55 C1 Prompt 30<br>*"I've noticed people do stand-up more in the office when doing skype calls and things"* P51 C1 Prompt 60
Barriers to engagement/ compliance	Workload, job tasks, location and perceived organizational expectations	Organizational and Environmental	"Too busy, Yeah, just too busy sounds a bit of a lame excuse but do you know when you're doing something doing and you're right in the middle of it and it goes 'buzz' and you get quite frustrated, I just want to concentrate what I'm concentrating on" P55 C1 P30<br>"I did have to turn mine off three weeks because my work just couldn't let me take time to stand up" P42 C2 P30
Acceptability and feasibility of 30 and 60 minute prompts	Task dependent	Organizational	"…so if it was just like in a departmental time meeting or even on Skype I'd stand up in Skype, no one can see me. So, when one of those calls but if there's an important meeting then I'd just ignore [the prompt]" P05 C1 P60
	Frequency of prompt	Interpersonal and organizational	*"Yeah, because those 30 minutes go really quickly. Yeah, you know, and although its very simple to do, when you know it is just like that but when you are kind of working and focusing, and times going quick you just think. Yeah, you kind of have a little bit of a sigh really when you see it pop up"* P44 C2 P30<br>*"I think that the 60 minute duration was good for me I don't think I would've liked a shorter duration the 30 minute one"*. P05 C1 P60<br>*"So I think it's just to keep me aware that I need to get up and move, maybe not every half an hour maybe an hour every hour would be better, as you say It flies by every 30 minutes my goodness"* P10 C1 P30
Organizational and cultural expectations around sitting behaviors at work	Challenging perceptions around 'acceptable' working behaviors in the workplace	Organizational and intrapersonal	*"The danger of* [frequently breaking up sitting time at work] *though is that people might feel conscious that if they were, I was spending too long too long away from their desks they might be seen as a slacker for not working*." P55 C1 Prompt 30

			"I think you tend to think you're being paid to work. So I focus on the work all the time without thinking about getting up […] I'm there to work". P49 C2 Prompt 30<br>*"I think its made it less of an odd behavior. Before If someone stood up you'd think 'oh why is he stood up to do his skype call' whereas now maybe the whole idea of standing up at your desk is not that weird. So its acceptable"* P55 C1 P30
Tailoring future e-health interventions to increase compliance	E-health interventions which are not a one size fits all.	Interpersonal	*"… if you had a thing like this* [smartphone application] *that was going to buzz you to stand if that was going to say that you have achieved 5,000 steps whats good for your health. Generally, the data show that you've done something that feeds in things like that perhaps would be more beneficial if it was automated somehow."* P 44 C2 P30
Diminishing engagement with e-health prompts over time	Lack of physical or psychological incentives and reduced motivation over time	Interpersonal	*"I think if I thought 'oh okay I feel much better for that' I would have carried on doing it, but it didn't seem to make any difference to how I felt physically or emotionally. So I was getting nothing from it I wasn't even getting emotional reward about saying 'Oh well done you've worked hard now you get a break' no you get nothing."* P55 C1 P30<br>*I think in the beginning, I was hundred percent I was as you said, I want to do it every half hour and I was like 'right I've got to do it, I've got to go and have a walk' and I'd make myself walk around the printer and back but I think as the weeks went on it kind of slid off […] Maybe the last 5 or 6 weeks maybe, I'd think 'okay I'll do it now in a minute'* P10 C1 P30
	Persistent or fluctuating workload pressures	Organizational	*"What I found towards the end was that I was telling it, don't bother me for 90 minutes, and then, you know, so moving on a bit, and then that 90 minutes to take it to the end of the day. And so then, I wouldn't have to bother with it until the next day"* P48 C2 P30

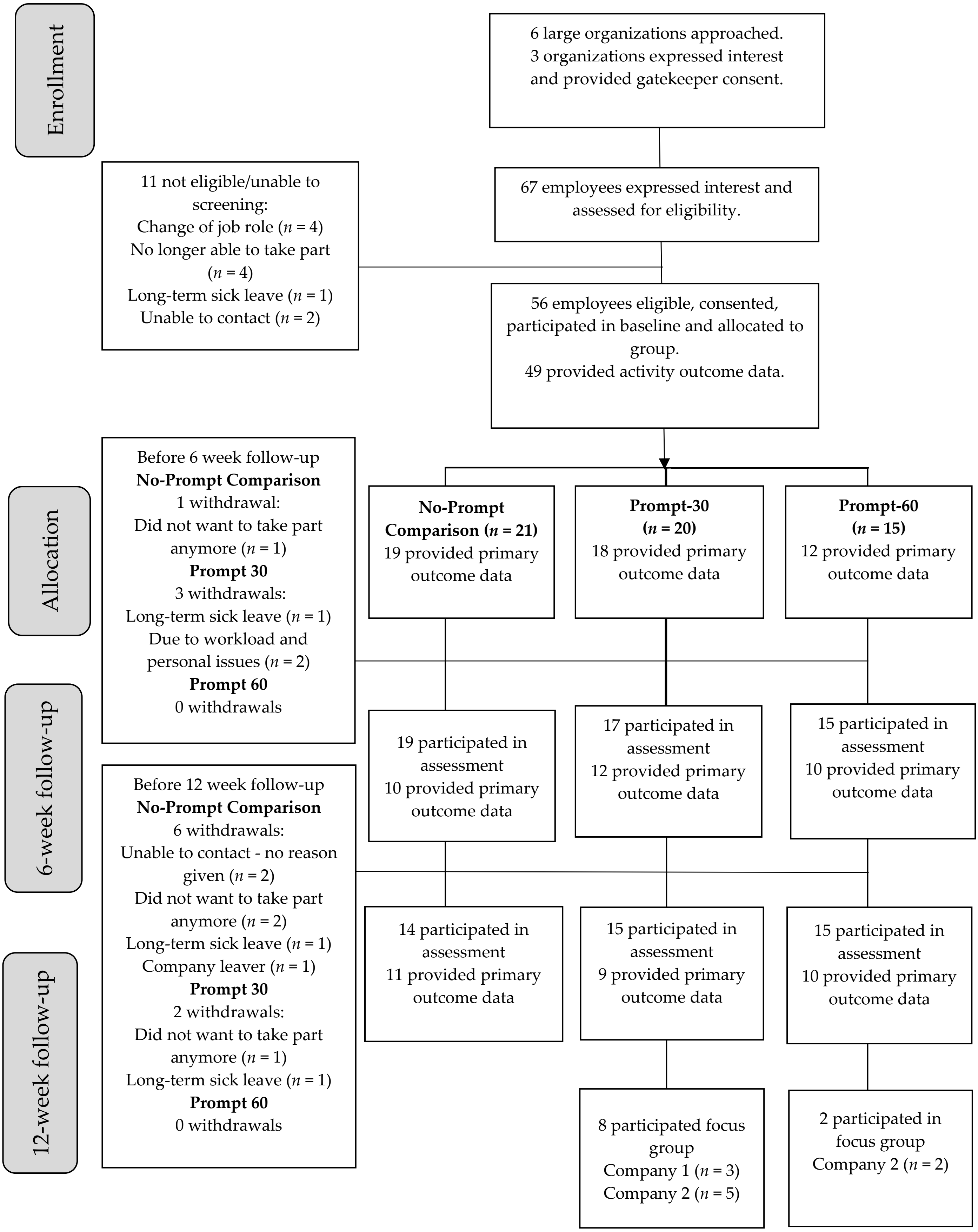

Figure: Supplementary materials 1. Consort flow diagram of enrolment, allocation, follow-up and analyses.

Figure: Supplementary materials 2: Completed Prompt and Review forms over the 12-week intervention.

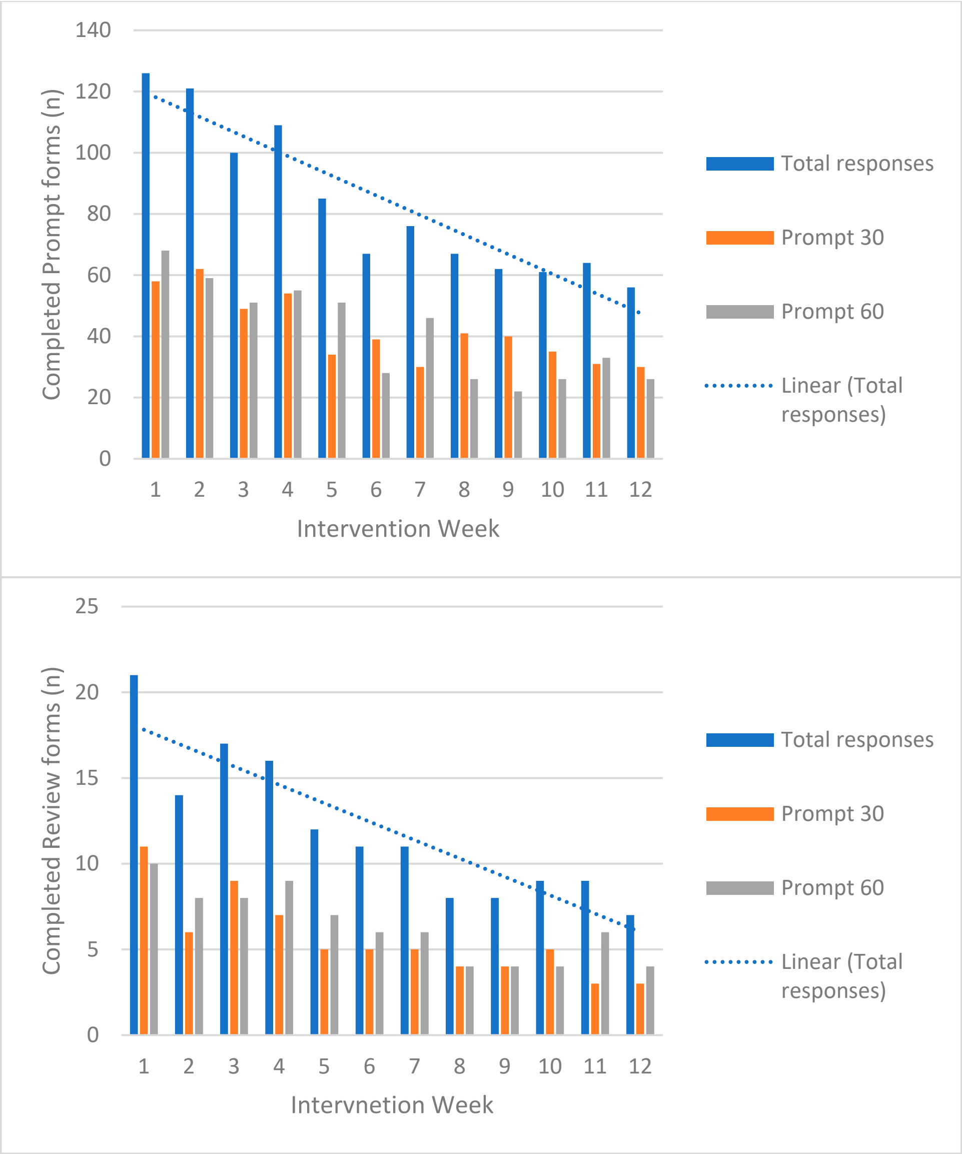

Supplement: Supplementary file 1 [file ijerph-18-09627-s001.zip › ijerph-1352348-SI.pdf]
